# From Micro to Marvel: Unleashing the Full Potential of Click Chemistry with Micromachine Integration

**DOI:** 10.3390/mi16060712

**Published:** 2025-06-15

**Authors:** Zihan Chen, Zimo Ren, Carmine Coluccini, Paolo Coghi

**Affiliations:** 1 School of Pharmacy, Macau University of Science and Technology, Macau 999078, China; zhchen@must.edu.mo (Z.C.); 2230028575@student.must.edu.mo (Z.R.); 2 Institute of New Drug Development, College of Medicine, China Medical University, No. 91 Hsueh-Shih Road, Taichung 0402, Taiwan; 3 State Key Laboratory of Quality Research in Chinese Medicine, Macau University of Science and Technology, Macau 999078, China

**Keywords:** click reaction, 1,2,3-triazole, drug discovery, microrobots, MEMS (microelectronic mechanical system)

## Abstract

Micromachines, small-scale engineered devices prepared to carry out exact tasks at the micro level, have garnered great interest across different fields such as drug delivery, chemical synthesis, and biomedical applications. In emerging applications, micromachines have indicated great potential in advancing click chemistry, a highly selective and efficient chemical technique widely applied in materials science, bioconjugation, and pharmaceutical development. Click chemistry, distinguished by its rapid reaction rates, high efficiency, and bioorthogonality, serves as a robust method for molecular assembly and functionalization. Incorporating micromachines into click chemistry processes paves the way for precise, automated, and scalable chemical synthesis. These tiny devices can effectively transport reactants, boost reaction efficiency through localized mixing, and enable highly exact site-specific modifications. Moreover, micromachines driven by external forces such as magnetic fields, ultrasound, or chemical fuels provide exceptional control over reaction conditions, significantly enhancing the selectivity and efficiency of click reactions. In this review, we explore the interaction between micromachines and click chemistry, showcasing recent advancements, potential uses, and future prospects in this cross-disciplinary domain. By leveraging micromachine-supported click chemistry, scientists can surpass conventional reaction constraints, opening doors to groundbreaking innovations in materials science, drug discovery, and beyond.

## 1. Introduction

The intersection of micromachines and click chemistry symbolizes a novel frontier in chemical synthesis, biomedical applications, and materials science. From a micromachines perspective, it involves the construction of microarchitectures capable of autonomous or controlled movements, which significantly enhance reaction efficiency, selectivity, and local catalysis. Click chemistry serves as an ideal domain for the integration of micromachines, characterized by rapid, high-yield responses and minimal by-product formation. This review explores the synergies between these technologies, their underlying mechanisms, applications, advantages, and future potential.

## 2. Fundamentals of Click Chemistry

**Scheme 1:**
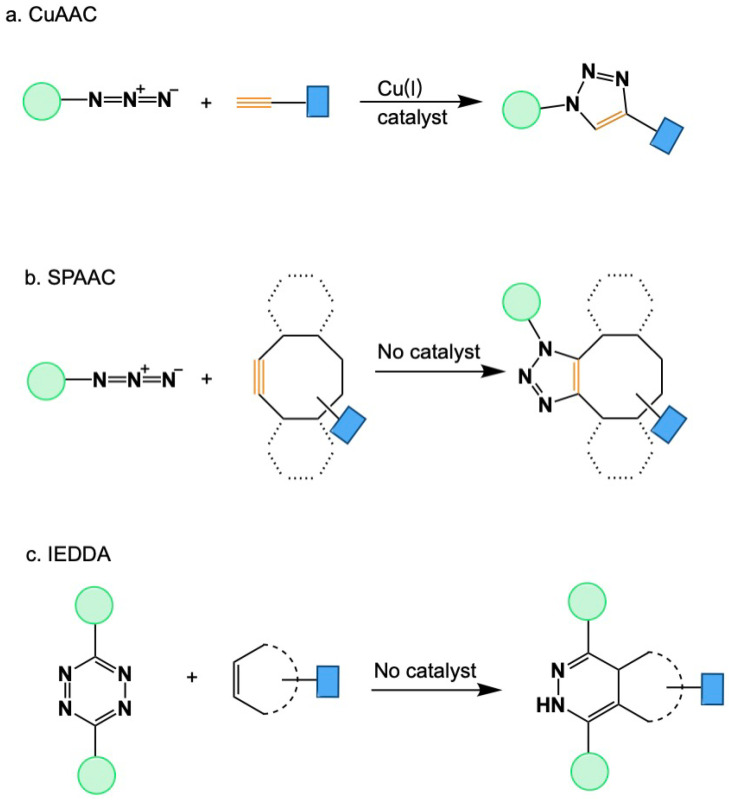
(**a**) CuAAC: copper-catalyzed azide–alkyne cycloaddition click reaction. (**b**) SPAAC: catalyst-free strain-promoted azide–alkyne cycloaddition. (**c**) IEDDA: inverse electron-demand Diels–Alder reaction.

Nobel Prize winner K. Barry Sharpless’s pioneering work created click chemistry, which is a paradigm shift in chemical synthesis that emphasizes efficient [1], selective [2], and biocompatible responses [3] under mild conditions with extraordinary fidelity [4,5]. This revolutionary approach has transformed molecular assembly by replacing traditional multi-step processes with simple, modular reactions that yield near-quantitative products with minimal byproducts [6,7]. The success of click chemistry lies in its ability to combine rapid reaction kinetics with special specificity, which makes it essential in different fields, including biomedical engineering [8], materials science [9], and drug development [10,11]. The centrality of this approach is the use of a variety of stable reactions, including copper-catalyzed azide–alkyne cycloaddition (CuAAC) [12], strain-promoted azide–alkyne cycloaddition (SPAAC) [13], and tetrazine ligation [14], and the excellent characteristics of rapid kinetics, near-quantitative yields, and exceptional biorthogonality, which often occur (Scheme 1). These reactions are particularly significant in the ability to perform efficiently in complex biological environments without disrupting natural biochemical processes [15].

**Scheme 2:**
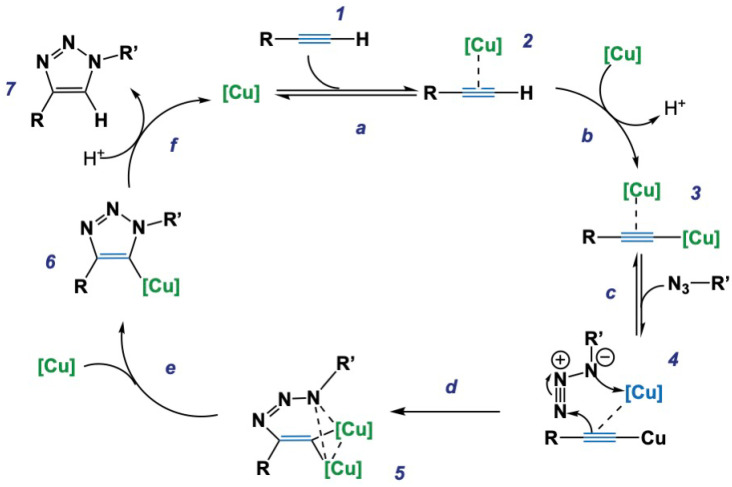
Schematic diagram of the catalytic cycle of the CuAAC reaction, Proceeding through Steps a–f, involving chemical species **1**–**7**.

The copper-catalyzed azide–alkyne cycloaddition (CuAAC) proceeds through a well-defined catalytic cycle initiated by Cu(I) coordination to a terminal alkyne, forming a copper acetylide complex (RC≡CCu) [12]. This coordination dramatically reduces the alkyne’s pKa (from ~25 to ~7), enabling deprotonation under mild basic conditions (e.g., amines) to generate the nucleophilic copper acetylide. The key cycloaddition step involves attack of this intermediate on the terminal nitrogen of an organic azide (R’-N₃), progressing via a six-membered copper-bound transition state (Cu-N-N-C≡C) that orchestrates a concerted [3+2] cyclization [16] (Scheme 2).

Here, the N-N bond of the azide cleaves while new C-N bonds form, yielding a 1,2,3-triazole ring. Crucially, Cu(I) not only accelerates the reaction by ~10^7^-fold but also enforces exclusive 1,4-regioselectivity (uncatalyzed variants produce 1,5-isomers). Catalytically active Cu(I) can be supplied in situ by reducing Cu(II) with ascorbate or using pre-stabilized ligands such as TBTA. The reaction tolerates aqueous media, ambient temperatures, and oxygen, making it ideal for bioconjugation (e.g., protein labeling), drug discovery, and polymer chemistry. Its robustness and orthogonality underpin its status as a quintessential “click” transformation [17]. The CuAAC reaction, for instance, forms stable triazole linkages in minutes with yields exceeding 95% [18,19], while the copper-free SPAAC variant enables safe labeling of biomolecules in living systems [20]. Tetrazine ligation stands out for its ultrafast kinetics, completing reactions in seconds for real-time imaging applications [21,22,23].

The strain-promoted azide–alkyne cycloaddition (SPAAC) reaction harnesses the inherent ring strain of cyclooctynes (typically 18–26 kcal/mol) to drive a copper-free [3+2] cycloaddition with azides. The distorted C≡C bond angle in cyclooctynes (e.g., DBCO, BCN) significantly activates the alkyne for direct reaction with azides without metal catalysis. This mechanism includes the interaction between the π orbit of the strained alkyne and the LUMO of the azide, resulting in a coordinate transition state that collapses to 1,4-disubstituted 1,2,3-triazole. SPAAC has a moderate to fast reaction rate (k^2^ ≈ 0.1–10 M^−1^s^−1^) and has a unique 1,4-regioselectivity, which provides many advantages for metal-sensitive applications such as live cell labeling and in vivo imaging. The release of ring strain energy provides substantial driving force (−15 to −20 kcal/mol), while the absence of copper eliminates potential cytotoxicity concerns. This bioorthogonal reaction has proven particularly valuable for labeling biomolecules in complex biological systems where traditional CuAAC would be problematic. The development of various cyclooctyne derivatives (e.g., DIBAC, BARAC) with tuned reactivity and water solubility has further expanded SPAAC’s utility in chemical biology and drug discovery. Its compatibility with physiological conditions and ability to proceed without exogenous catalysts make it indispensable for studying dynamic biological processes in native environments [24,25].

Bioorthogonality permits operation in complex biological milieu, opening doors to in vivo applications previously considered unfeasible [26,27,28]. The efficiency of click chemistry enables rapid, high-yield transformations even in the confined spaces characteristic of microscale environments [29]. In this context, Sulfur(VI) Fluoride Exchange (SuFEx) represents a powerful class of click chemistry first developed by Sharpless and colleagues [29].

The mechanism involves nucleophilic substitution at the hexavalent sulfur (S^6+^) center, leveraging the high electrophilicity of S(VI)–F bonds while maintaining stability under physiological conditions [30]. This robust conjugation technique exploits the unique reactivity of S(VI)-F bonds, which exhibit exceptional stability in biological media, undergo rapid and selective reactions with nucleophiles (e.g., phenols, amines), and deliver near-quantitative yields under mild conditions.

Recent advances demonstrate how enzyme-powered micromotors can perform site-specific thiol–ene click reactions [31] for polymer functionalization, while AI-guided microswarms optimize reaction networks for continuous-flow synthesis, highlighting the transformative potential of this integration [32].

However, significant challenges remain in fully realizing the potential of micromachine-assisted click chemistry [33]. Achieving biocompatibility in micromachine materials poses a key challenge for medical applications, necessitating the use of non-toxic and degradable components. Meanwhile, scaling these systems for industrial use requires innovative solutions to preserve precision while enhancing production throughput [33].

## 3. Micromachines: Design and Functionality

Micromachines, also known as microelectromechanical systems (MEMS), are micro-devices that integrate mechanical structures, sensors, actuators, and control electronics into a single chip. These systems usually work on micrometer scales and are designed in some way to perform professional tasks in different fields, including biomedical engineering, chemical analysis, and environmental sensing [34].

The design of micromachines is function-oriented, with the architecture tailored to the specific application, whether that is manipulating fluids, detecting biomolecules, or providing localized actuation [35]. Key components often include actuators, which convert energy into mechanical motion. Common types are electrostatic, piezoelectric, thermal, and magnetic actuators. Each actuator type offers distinct advantages: electrostatic actuators are fast and energy-efficient, while piezoelectric ones offer high precision and are well-suited for sensing and actuation at small scales [36]. Recent reviews highlight the integration of these actuators into biomedical devices, emphasizing their role in applications such as minimally invasive surgery and implantable systems [35].

Sensor integration is critical for autonomous or feedback-based operation. Sensors within micromachines can detect pressure, temperature, pH, or even specific biochemical markers. For instance, biosensors embedded in MEMS platforms have enabled real-time monitoring in diagnostic applications and environmental monitoring [37]. These sensors often work in tandem with actuators to create closed-loop control systems that enhance accuracy and responsiveness.

The selection of materials plays a fundamental role in determining the properties of micromechanics. Silicon is the most commonly used material due to its mechanical robustness and compatibility with micromachining processes. However, depending on the use and requirements, other materials such as polymers (e.g., PDMS), metals (e.g., gold, platinum), and ceramics are increasingly used for their many advantages such as biocompatibility, flexibility, or corrosion resistance [38].

The preparation of micromachines is heavily based on technologies in the semiconductor industry, including photolithography, chemical etching, and thin-film deposition [39]. The latest developments introduce soft lithography and 3D micro-printing technologies that can create more complex structures and improve the performance of micromachines in biomedical and chemical applications [40,41]. These techniques have enabled scalable and precise fabrication, paving the way for the commercial production of MEMS-based devices [42].

Functionally, micromachines are engineered for high autonomy and precision. In microfluidics, they can manipulate picoliter-scale fluid volumes, facilitating lab-on-a-chip systems used in diagnostics and drug screening [43]. Similarly, in biomedical applications, implantable micromachines are being developed for tasks such as controlled drug release, real-time biosensing, and minimally invasive therapeutic interventions [44].

Despite significant progress, several challenges remain. Powering micromachines, ensuring long-term operational stability, and achieving robust wireless communication are key barriers, particularly for in vivo or remote applications. Nevertheless, continued advancements [45] in materials science, microfabrication techniques, and system integration are rapidly overcoming these obstacles [46,47]. As a result, micromachines are poised to become foundational tools in next-generation healthcare, smart manufacturing, and precision research environments.

## 4. Integration of Micromachines with Click Chemistry

The true power of click chemistry emerges when integrated with micromachine technology, where these tiny engineered devices overcome traditional limitations by providing precise spatiotemporal control over reaction environments [48,49].

Click chemistry, known for its reliability, simplicity, and versatility, allows for the rapid and efficient formation of covalent bonds between functional groups under mild conditions [45,50]. When integrated with micromachines—miniaturized devices capable of performing complex microscopic-scale operations—this synergy enables breakthrough applications in targeted drug delivery, advanced diagnostics, and precision environmental monitoring [51]. The integration enables micromachines to undergo specific and well-controlled chemical reactions at precise locations within the body or other environments, ensuring that therapeutic agents or sensors are activated or released only when and where needed. Furthermore, the adaptability of click chemistry allows for the functionalization of micromachines with various bioactive molecules, providing a platform for creating biohybrid systems that can interact with biological systems in a highly controlled manner [52]. This powerful combination of technologies not only enhances the capabilities of micromachines but also broadens their potential applications [53], from advanced biomedical interventions to environmental cleanup efforts.

### 4.1. Enhanced Reaction Kinetics

A key advantage of click reactions lies in their rapid and efficient reaction kinetics, which enable bioorthogonal modifications to occur in real time under mild conditions [53,54]. Enhanced reaction rates, particularly in strain-promoted or catalyst-free systems, are essential for applications where micromachines must operate dynamically within complex biological environments [55]. Fast and stable reactions ensure reliable surface modification, controlled cargo release, and real-time sensing, thereby expanding the functional scope of micromachine-assisted systems.

**Figure 1:**
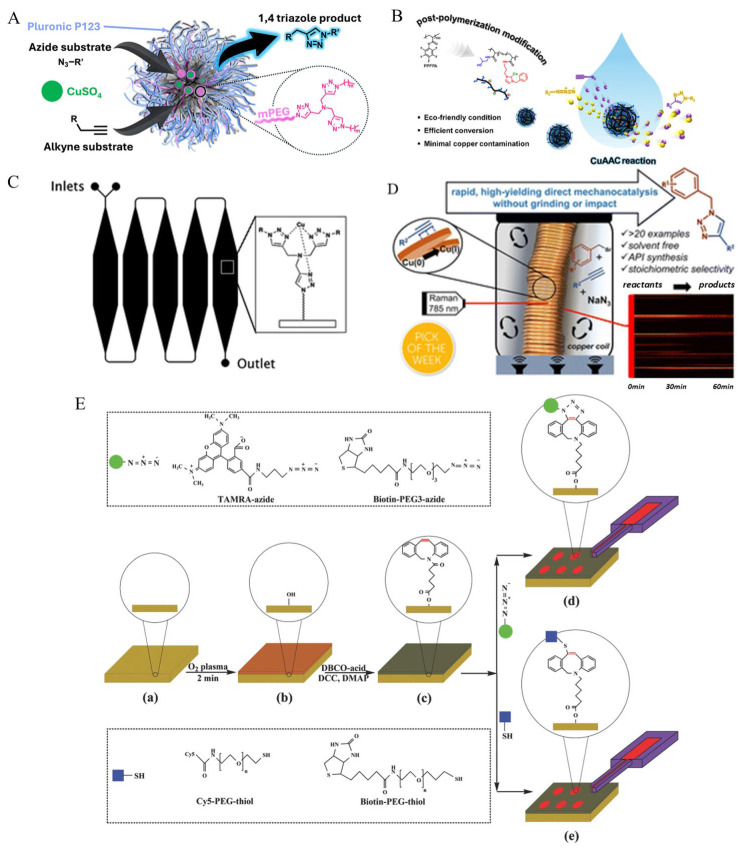
(**A**) Mechanism of the CuAAC reaction catalyzed by functionalized PEG with Pluronic P123 co-assembled MNRs. (**B**) Mechanism of poly(N-(2-hydroxypropyl)acrylamide)-ran-poly(N-(3-(1-benzylimidazolium-3-yl)propyl)acrylamide) PHPAM76-ran-PILAM24 (Cu(I)) micellar catalysts to accelerate the CuAAC reaction. (**C**) Tris-(benzyltriazolylmethyl)amine derivative-Cu(I) microfluidic “click chips”. (**D**) Mechanism of the resonant acoustic mixing catalysed CuAAC reaction. (**E**) Azide-alkyne and thiol-alkyne click reactions on DBCO acid-functionalized hydroxyl-terminated glass, (**a**) bare glass; (**b**) hydroxyl-terminated glass; (**c**) DBCO-terminated glass; (**d**) treatment of the DBCO-terminated surface with TAMRA-azide and biotin-PEG3-azide; (**e**) treatment of the DBCO-terminated surface with Cy5-PEG-thiol and biotin-PEG-thiol. Reprinted with permission from [56,57] Copyright 2024, 2025 American Chemical Society. Reprinted with permission from [58,59]. Copyright 2014, 2023 RSC Pub. Reprinted with permission from [60] Copyright 2025 John Wiley and Sons.

Micromachines can actively transport reactants and catalysts to reaction sites, significantly accelerating reaction rates. Micellar nanoreactors (MNRs) were engineered by incorporating dendritic amphiphiles (DTAs) into Pluronic P123 micelles, and they facilitated the copper(I)-catalyzed alkyne-azide cycloaddition (CuAAC) reaction by concentrating reactants within the micelles, thereby significantly reducing reaction times from several hours to approximately 20 min (Figure 1A) [56]. While the study does not involve micromachines, the principles demonstrated could inform future designs of micromachine systems that require localized catalytic activity or bioorthogonal functionalities.

The polymeric micellar nanocatalysts can be considered as nanoscale catalytic systems that perform specific chemical transformations in aqueous environments. Their self-assembled structure and catalytic functionality align with certain aspects of micromachine behavior, particularly in the context of performing targeted tasks at the nanoscale.

A compelling illustration of this concept is found in the synthesis of a random copolymer via post-polymerization modification of poly(pentafluorophenyl acrylate) (PPFPA) with 1-amino-2-propanol and other nucleophilic agents, followed by alkylation and copper insertion to produce a polymer-supported copper(I) catalyst, PHPAM76-ran-PILAM24(Cu(I)). This catalyst forms micelles in water and effectively catalyzes copper-catalyzed azide–alkyne cycloaddition (CuAAC) reactions. With just 1 mol% Cu loading, it enables high yields (>95%) and complete conversion at room temperature within 1–4 h (Figure 1B) [57].

Microfluidic devices are miniaturized systems designed to handle and manipulate fluids, typically in volumes ranging from microliters to picoliters, through channels just tens to hundreds of micrometers wide. These systems with immobilized copper(I) catalysts have been developed to facilitate click reactions. These “click chips” enable the efficient and rapid conjugation of molecules such as fluorescent dyes to peptides under continuous flow conditions. The immobilized catalysts on the chip surfaces enhance reaction rates, allow for multiple reuses of the device, and improve immobilization efficiency while reducing the diffusion time of reagents to the active catalyst sites (Figure 1C) [58].

The capacity of micromachines to induce localized turbulence enhances the mixing efficiency, thereby accelerating reaction rates. A novel approach employs resonant acoustic mixing to enable direct mechanocatalysis, eliminating the requirement for milling media and bulk solvents. In this approach, a copper wire coil catalyzes the azide–alkyne click coupling reaction under vibrational mixing. This solvent-less method offers a scalable alternative to traditional CuAAC, potentially transforming synthetic strategies by minimizing solvent use and simplifying processes (Figure 1D) [59].

This acceleration is crucial in industrial applications where rapid chemical synthesis is essential, as well as in biomedical settings where real-time reactions can improve therapeutic efficiency. For instance, a recent study compared two catalyst-free click chemistry approaches, azide–alkyne and thiol–alkyne reactions, for immobilizing microarrays on functionalized glass surfaces using microchannel cantilever spotting (µCS). The research highlights the role of the microfluidic design in enhancing the reaction efficiency, which is influenced by factors such as flow dynamics and mixing performance within the microchannels [60].

A published study introduced a microfluidic system that exploits spontaneous symmetry breaking in mesoscale turbulence to perform work. The researchers demonstrated that such active micromachines could induce chaotic advection, leading to enhanced mixing and improved reaction rates [61]. The research highlighted in reviews [62,63] examined various micromixer designs, noting that both passive and active micromixers can significantly improve mixing efficiency. Active micromixers, in particular, utilize external forces to induce turbulence and chaotic flows, thereby enhancing mixing performance in microfluidic devices.

### 4.2. Selective and Localized Reactions

**Figure 2:**
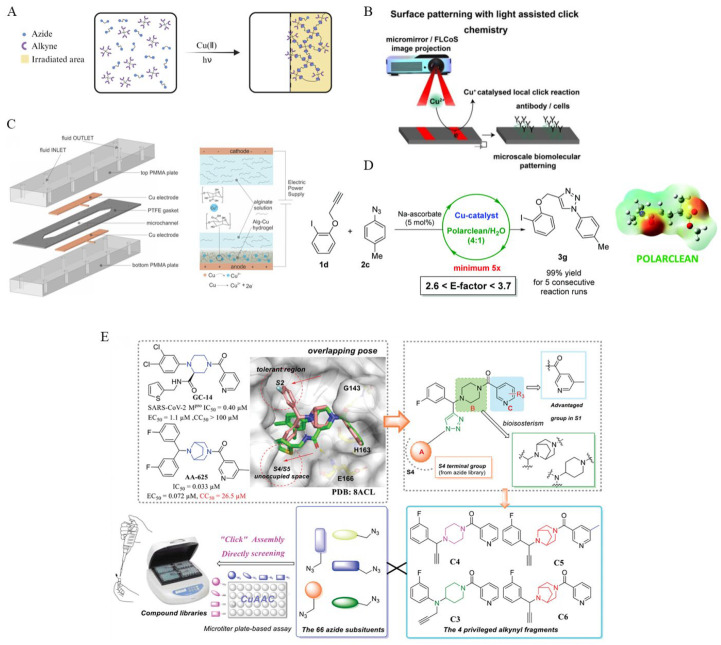
(**A**) Photoinitiated Cu(II) catalyst-catalyzed CuAAC reaction. (**B**) Light-assisted click chemistry for biomolecular surface patterning. (**C**) Construction of Alg-Cu hydrogel catalyst. (**D**) Polarclean/water-phase microfluidic metal catalyst catalyzes the CuAAC reaction. (**E**) Synthesis of SARS-CoV-2 M^pro^ inhibitors by click chemistry using micromachines and microfluidics. Reprinted with permission from [66]. Copyright 2020 John Wiley and Sons. Reprinted with permission from [71]. Copyright 2024 Ramšak et al. Frontiers. Reprinted with permission from [72]. Copyright 2018 RSC Pub. Reprinted with permission from [77]. Copyright 2024 Yang et al. Advanced Science published by Wiley-VCH GmbH.

Micromachines enable the spatial control of click reactions, allowing for site-specific modifications on surfaces or within complex biological systems. Adzima et al. demonstrated the precise spatial and temporal control of click reactions by employing photoinitiated reduction of Cu(II) to Cu(I), enabling site-specific surface modifications using standard photolithographic techniques. Their photochemical approach effectively generates the active Cu(I) catalyst in situ, offering a robust strategy for controlled CuAAC reactions with high spatial resolution (Figure 2A) [64]. A review [65] highlights the application of bioorthogonal click chemistry in creating complex cell culture scaffolds with spatial and temporal control over biochemical cues. This property is particularly useful in tissue engineering and targeted drug conjugation, where localized chemical modifications are necessary.

By guiding micromachines to specific target locations using external stimuli such as magnetic fields or light, researchers can confine click reactions to desired areas. This minimizes off-target effects and enhances modification precision [66]. Gautam et al. presented a light-assisted click chemistry approach for the chemical patterning of semiconducting surfaces. By illuminating specific regions of an electrode with visible light, localized electrogeneration of the Cu(I) catalyst was achieved, enabling site-specific click reactions. The surface modifications directly corresponded to a user-defined 2D light pattern projected onto a silicon photoelectrode. This mask-free, parallel process allows the simultaneous functionalization of distinct surface regions with various molecules. As a proof of concept, azido-poly(ethylene glycol) (PEG) molecules were covalently patterned onto alkyne-functionalized electrodes. Furthermore, by tailoring the exposed functional groups of the clicked PEGs, the spatially controlled adhesion of antibodies and cells was successfully demonstrated. This structured light strategy significantly reduces the patterning time to just a few hours, offering a rapid and versatile alternative to conventional masked techniques (Figure 2B) [66]. Other researchers have developed a continuous-flow microfluidic reactor that utilizes electrostatic fields to catalyze azide–alkyne cycloaddition reactions. This system demonstrates superior catalytic performance compared to traditional Cu(I)-catalyzed methods, offering enhanced reaction rates and selectivity [67]. Magnetic micromotors can deliver Cu catalysts for localized CuAAC reactions while minimizing cytotoxic effects, addressing one of the major challenges in biomedical applications [68,69].

### 4.3. Reagent Consumption and Environmental Impact

By optimizing reagent delivery and minimizing waste, micromachines contribute to the sustainability of click chemistry applications [10]. This is particularly relevant in biomedical applications where minimizing exposure to excess reagents is crucial for biocompatibility [70]. Efficient reagent utilization also lowers production costs and reduces environmental impact.

A 2024 study by Ramšak et al. introduced copper–alginate hydrogels within microfluidic devices to catalyze [3+2] dipolar cycloaddition reactions, a type of click chemistry. Utilizing biocompatible and biodegradable alginate as a catalyst support, the system achieved high regioselectivity and conversion rates. The microfluidic setup allowed for the precise control over reaction conditions, enhancing reaction kinetics and mass transfer efficiencies. Compared to traditional batch methods, this approach reduced solvent usage and waste generation, aligning with green chemistry principles (Figure 2C) [71].

Research by Vaccaro et al. demonstrated a continuous flow copper-catalyzed azide–alkyne cycloaddition (CuAAC) process with minimized metal contamination. The flow system enabled efficient reactions with reduced environmental impact, highlighting the benefits of microfluidic technologies in sustainable chemical synthesis and making it relevant to the development of micromachine-based chemical processes (Figure 2D) [72].

A review by Chen et al. discussed the integration of microflow chemistry with electrification techniques, such as microwaves and plasmas, to enhance sustainability in chemical manufacturing. The precise control offered by microfluidic systems leads to an improved yield and productivity while minimizing waste and energy consumption [73].

Furthermore, micromachines can function as mobile catalytic microreactors, enabling continuous catalyst transport and regeneration within reaction systems. This capability significantly improves process sustainability while enhancing reaction efficiency, which is particularly valuable in industrial-scale manufacturing where resource optimization is paramount [74].

Researchers have utilized droplet microfluidics to fabricate hyaluronic acid (HA) microgels through various click reactions, strain-promoted azide–alkyne cycloaddition (SPAAC), and UV-initiated thiol–ene reactions [75]. This approach significantly reduces the amount of reagents required compared to bulk synthesis and allows for the creation of microgels with tailored properties for applications in cell biology and biotechnology.

### 4.4. Automation and Programmability

Micromachines can be interfaced with microfluidic platforms [76] and lab-on-a-chip devices, enabling the automated and high-throughput synthesis of click chemistry-derived compounds (Figure 2E) [77]. This can improve reproducibility and scalability in industrial and pharmaceutical environments. Through engineered programmability, micromachines can autonomously regulate reaction systems by dynamically controlling key parameters including the reactant concentration, temperature, and catalyst activity in real time. This advanced automation enhances process efficiency while eliminating human intervention, establishing micromachine-enabled click chemistry as a transformative platform for precision medicine and advanced materials engineering.

Research by Wang et al. demonstrated a closed-loop catalytic nanoreactor system built on a transistor. This setup allows for the real-time modulation of enzyme activity through electrical signals, effectively turning reactions “on” or “off” and adjusting catalytic efficiency [78].

A study by Zhang et al. reported the development of smart microreactors equipped with responsive catalytic nanoparticles. These reactors can self-regulate exothermic reactions by adjusting the catalytic activity in response to temperature changes, ensuring safety and efficiency without manual intervention [79].

These examples illustrate the potential of programmable micromachines and microreactor systems to create self-regulating environments for click chemistry. By enabling real-time adjustments and minimizing human error, these technologies hold promise for advancing precision medicine and material fabrication.

### 4.5. Micromachine-Assisted Click Chemistry in Complex Biological Environments

The integration of micromachine-assisted click chemistry within dynamic biological milieus such as tumors, inflammatory sites, or infected tissues requires precise engineering to overcome microenvironmental challenges while maintaining reaction efficiency and selectivity. These dynamic microenvironments present multiple challenges that demand innovative engineering solutions. Chemically, micromachines must operate under conditions of hypoxia, acidic pH (as low as 6.5 in tumors) [80], and high concentrations of reactive oxygen species [81] that can degrade catalysts or reactants. Biologically, they encounter enzymatic degradation (particularly from esterases), protein fouling, and nonspecific cellular uptake that can interfere with their function [82]. Physically, heterogeneous tissue stiffness, viscous mucus barriers (especially in gastrointestinal or pulmonary inflammation), and fluid shear forces further complicate reliable operation. While phenomena such as the enhanced permeability and retention effect facilitate micromachine accumulation in tumors, the dense extracellular matrix in these tissues often limits their penetration and uniform distribution.

To address these challenges, researchers have developed sophisticated micromachine designs that incorporate localized reaction control, environmental sensing capabilities, and enhanced targeting mechanisms. Encapsulation strategies using pH-responsive polymers protect sensitive click chemistry reactants (such as azides and strained alkynes) until they reach the target site [83], while immobilized catalysts such as BTTAA-ligated Cu(I) complexes or enzyme mimics enable in situ reaction activation. Stimuli-responsive systems [84] take advantage of pathological conditions, with pH-sensitive SuFEx reactions [29] and ROS-activated tetrazine ligation providing selective activation precisely where needed. Advanced targeting approaches, including ligand–receptor interactions (using folate or RGD peptides) and external magnetic guidance, further improve localization at disease sites.

These engineering solutions have demonstrated remarkable success in real-world applications. In tumor environments, porous silicon micromachines loaded with dibenzocyclooctyne and coated with hyaluronidase have achieved five-fold improvements in tumor labeling efficiency compared to passive diffusion approaches by simultaneously degrading the extracellular matrix and performing bioorthogonal click reactions. Similarly, in inflammatory conditions such as arthritis, magnesium-based Janus micromotors equipped with tetrazine surfaces [85] have enabled the real-time monitoring of neutrophil activity through ratiometric fluorescence signaling. These examples highlight how the integration of smart material design with click chemistry principles can overcome biological barriers while providing precise diagnostic and therapeutic capabilities.

## 5. Recent Advances and Future Prospects of Micromachine-Assisted Click Chemistry in Biomedical Applications

The combined use of micromachines and click chemistry has been explored in various fields, including the following:

### 5.1. Drug Delivery and Bioconjugation

One of the most promising applications is in targeted drug delivery, where micromachines facilitate the attachment of therapeutic molecules to biological targets [86]. This has been demonstrated in cancer therapy, where drug-loaded micromachines selectively bind to tumor cells through bioorthogonal reactions [87]. Micromachines enable site-specific drug conjugation, allowing researchers to deliver higher drug concentrations to targeted areas while significantly reducing systemic toxicity [88]. This micromachine technology allows exact labeling of biological markers for imaging, leading to more accurate diagnoses and tailored treatment options [89].

**Figure 3:**
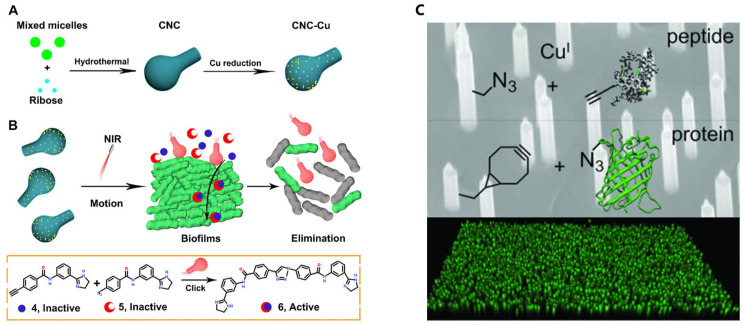
(**A**) Synthesis of CNC-Cu. (**B**) Mechanism of CNC-Cu catalyzing targeted drug synthesis in biofilms. (**C**) Click chemistry functionalization of semiconductor nanowires. Reprinted with permission from [90]. Copyright 2022 American Chemical Society. Reprinted with permission from [94]. Copyright 2015 John Wiley and Sons.

While direct examples of self-propelled micromachines employing click chemistry for drug delivery remain limited, their integration represents a highly promising research frontier. Future advances will likely combine the autonomous navigation of micromachines with the precision of bioorthogonal click reactions to develop next-generation targeted drug delivery systems. The Cu(I)-catalyzed azide–alkyne cycloaddition (CuAAC) reaction serves as a cornerstone of bioorthogonal chemistry, offering high selectivity and stability. However, the delivery of synthetic bioorthogonal drugs into deep tissues and biofilms remains challenging due to biological barriers. To address this, Liu et al. developed an NIR light-responsive carbonaceous nanocalabash (CNC) motor catalyst capable of autonomous motion and in situ drug synthesis within biofilms [90]. Under the radiation of the near-infrared laser, the CNC catalyst displays rapid autonomous motion and thus can penetrate deep into the biofilm layers. Once the bioorthogonal reactions are catalyzed, especially the Cu(I)-catalyzed azide–alkyne cycloaddition (CuAAC), it is possible to synthesize active therapeutic molecules in situ. The innovative platform improves drug penetration, destroys biofilms and effectively removes bacteria, and provides promising strategies for the bioorthogonal therapy of deep tissues (Figure 3A,B).

Key advantages of SuFEx [91] for micromachine systems include its versatile surface modification via SO_2_F groups that form stable covalent linkages with diverse materials (e.g., SiO_2_, polymers), excellent aqueous compatibility with efficient reaction kinetics in water and physiological buffers, and orthogonality to other click chemistries such as SPAAC and tetrazine ligation. Recent applications demonstrate SuFEx’s growing potential in microdevice biofunctionalization [29], stimuli-responsive surface engineering [92], and in situ micromachine assembly.

### 5.2. Biosensing and Diagnostics

Micromachines have been utilized in biosensing applications, where they selectively interact with biomolecules [93]. Li et al. highlighted the use of magnetic helical microswimmers functionalized with lipoplexes for targeted gene delivery. These microswimmers are designed to navigate through biological environments and deliver therapeutic payloads to specific sites. The robots utilize specific surface functionalizations (e.g., antibodies, peptides) to achieve targeting and enable the precise control over the delivery of genetic material to targeted cells, enhancing the efficacy of gene therapy while minimizing off-target effects. Moreover, the autonomous propulsion capability of micromachines through biological fluids enables rapid target detection and enrichment, significantly enhancing both the sensitivity and processing speed of diagnostic assays.

Furthermore, micromachines functionalized with click chemistry reagents could be designed to respond to specific biomarkers, allowing for the real-time monitoring of disease progression and treatment response [94]. In a study, vertical nanowires were functionalized using click chemistry to enable the precise attachment of biomolecules or therapeutic agents. These nanowires were engineered to interact with biological systems, facilitating targeted drug delivery, biosensing, or tissue engineering. The use of click chemistry ensured the efficient and specific functionalization of the nanowires, which could be considered a micromachine system when deployed for drug delivery or other biomedical tasks (Figure 3C).

### 5.3. Smart Materials and Surface Functionalization

Micromachines aid in surface modification and functionalization, enabling the creation of responsive materials, such as magnetic hydrogel microscrews and microrollers, which respond to changes in magnetic fields, temperature, and pH. These systems are fabricated using 3D printing techniques, allowing for precise control over their size and responsiveness, making them suitable for various biomedical applications [95].

Click chemistry allows for the rapid modification of polymer surfaces, contributing to advancements in antifouling coatings [96], biosensors [97], and tissue engineering scaffolds. By integrating micromachines into material synthesis, researchers could achieve dynamic surface changes that respond to environmental cues, leading to smart materials with adaptive properties [98]. These advancements have implications for a wide range of industries, from biomedical implants that resist biofouling to self-healing materials for structural applications [99].

### 5.4. Environmental Remediation

In environmental applications, micromachines functionalized with click chemistry reagents can selectively capture and neutralize pollutants. Heavy metal sequestration and microplastic degradation are emerging areas of research where these technologies could have a significant impact [100,101]. Micromachines can navigate contaminated water sources, actively binding to harmful substances and facilitating their removal. Additionally, click chemistry-based functionalization enables the design of highly specific pollutant-capturing agents, improving the efficiency of environmental cleanup efforts. The ability of micromachines to operate autonomously in complex environments makes them valuable tools for addressing pressing ecological challenges [101].

## 6. Challenges and Limitations

The development of energy-efficient autonomous micromotors that can sense and respond to chemical cues without external power sources presents both a technical challenge and an exciting research frontier [102]. Looking ahead, the convergence of click chemistry principles with increasingly sophisticated micromachine designs promises to revolutionize multiple scientific and technological domains. In precision medicine [103], bioorthogonal reactions coupled with targeted micromachines could enable breakthrough theranostic platforms capable of simultaneous diagnosis and treatment at the cellular level. For sustainable materials science, this integration may lead to self-assembling, self-repairing materials with precisely controlled properties. The programmability of click reactions combined with the automation potential of micromachines is creating new possibilities in molecular manufacturing that were previously unimaginable, setting the stage for a new era of smart, adaptive chemical systems that blur the boundaries between chemistry, engineering, and biology [104]. Despite their numerous advantages, the integration of micromachines and click chemistry presents several other challenges:

### 6.1. Biocompatibility and Toxicity

Many micromachine materials, particularly metal-based catalysts (e.g., copper in CuAAC), pose toxicity concerns.

Click chemistry is highly specific and efficient, and the potential cytotoxicity of copper ions requires the development of copper-free click chemistry alternatives (e.g., strain-promoted azide–alkyne cycloaddition, SPAAC) for safer biomedical integration. Meanwhile, ongoing research aims to improve the biocompatibility and biodegradability of micromachines [105] by employing enzyme-powered motors [106], biodegradable polymers, and bioinspired materials. These advances not only mitigate toxicity risks but also broaden the applicability of micromachine-assisted click chemistry in clinical diagnostics, targeted drug delivery, and in vivo imaging.

### 6.2. Control and Stability

The efficiency of click chemistry reactions depends on several critical factors, including reagent compatibility, reaction conditions, and catalyst toxicity, particularly in biological systems. While copper-based catalysts demonstrate high reactivity, their inherent cytotoxicity necessitates the development of catalyst-free alternatives. Furthermore, achieving the stable surface functionalization of micromachines under physiological conditions remains essential for reliable performance. Addressing these challenges is crucial for advancing micromachine applications in targeted therapies, diagnostic systems, and responsive materials. Achieving precise control over micromachine movement in complex environments remains a significant challenge, as highlighted in a review article [107]. This comprehensive study discusses the current state and future prospects of micro–nano robots, particularly those driven by physical fields such as magnetic, acoustic, and optical forces. The intersection and fusion of microelectronics has many applications, such as micro–nano processing, biology, physics, chemistry, machinery, automation, and other fields. This interdisciplinary approach is crucial to inspire new research perspectives and promote the development of micro–nanobots. Current research is focused on developing responsive micromachines that can be dynamically controlled using external stimuli such as light or magnetic fields [108,109]. One study explored how stimuli-responsive materials, such as thermoresponsive hydrogels and liquid crystal elastomers, can be integrated into microrobots to enable autonomous behaviors. These materials allow microrobots to adapt their shape and function in response to environmental cues, facilitating tasks such as targeted cargo release and navigation through complex biological environments [109].

### 6.3. Scalability and Cost

Although micromachine technology continues to make significant strides, the widespread adoption and integration of these systems into commercial click chemistry applications remain limited by high production costs and complex manufacturing processes. Current research and industrial efforts are focused on optimizing the fabrication techniques, scaling up production methods, and developing cost-efficient materials to make these advanced technologies more economically viable [110,111]. As these improvements progress, the potential for micromachines to revolutionize chemical synthesis, particularly in precision applications such as click chemistry, becomes increasingly attainable.

## 7. Toward the Next Generation of Applications of Micromachines in Click Chemistry

The future of integrating microscale robotic systems with click chemistry hinges on continued innovation in materials science, precision engineering, and advanced bioorthogonal chemistry. Some promising directions include the following:

### 7.1. AI-Driven Control and Machine Learning Approaches for Advanced Micromachines

**Figure 4:**
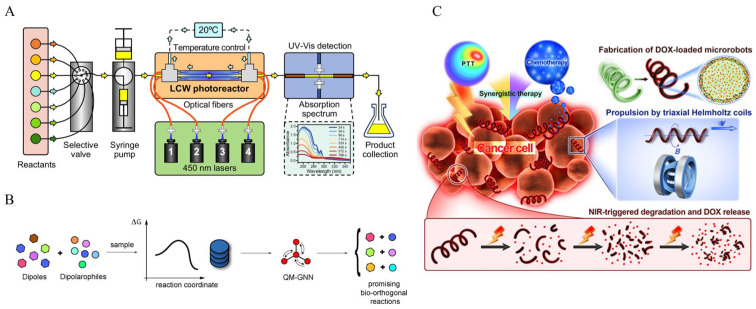
(**A**) Schematic diagram of the photocatalytic AI-assisted microfluidic reaction system. (**B**) Machine learning models for predicting bioorthogonal click reactions. (**C**) Magnetically driven degradable microrobots prepared using *Spirulina* for cancer therapy. Reprinted with permission from [112]. Copyright 2024 Lu et al. Springer Nature. Reprinted with permission from [118]. Copyright 2023 Stuyver et al. Chemistry-A European Journal published by Wiley-VCH GmbH. Reprinted with permission from [119]. Copyright 2019 American Chemical Society.

The integration of artificial intelligence (AI) and machine learning could enhance the precision and adaptability of micromachines, optimizing reaction conditions in real time. A study by Lu et al. introduced a roboticized, AI-assisted microfluidic platform capable of conducting up to 10,000 photocatalytic reactions daily [112]. This system autonomously adjusts variables such as the reactant concentration, temperature, and catalyst ratios, enabling rapid optimization and minimizing human error (Figure 4A). Such automation is pivotal for accelerating the discovery and development of new compounds in click chemistry. For a more direct exploration of AI-driven micromachines utilizing click chemistry, further research may be necessary, as this intersection is an emerging field.

Machine learning-guided computational screening is an increasingly powerful method used in various scientific fields, including materials science, chemistry [113], and drug discovery [114]. The goal of this method is to leverage machine learning algorithms to speed up and optimize the process of identifying promising materials, molecules, or compounds by simulating and analyzing large datasets in silico.

Machine learning has also been shown to offer a promising avenue to tackle these complexities in micro- and nanorobots [115] and in click chemistry [116,117].

Recently some authors presented a computational workflow that utilizes machine learning to discover new candidate bioorthogonal click reactions (Figure 4B) [118]. By predicting the activation and reaction energies, the model identifies a diverse set of potential reactions, expanding the toolkit for bioorthogonal chemistry. While the primary focus is on reaction discovery, the methodologies discussed could be applicable to the design and optimization of micromachines that rely on click chemistry for functionalization and control.

### 7.2. On-Chip Click Chemistry: Microfluidic Platforms for Next-Generation PET Tracer Development

The synthesis of PET tracers combined with micromachines and click chemistry represents a cutting-edge approach in molecular imaging and targeted drug delivery. Positron Emission Tomography (PET) tracers, typically radiolabeled compounds, allow for the non-invasive visualization of biological processes at the molecular level [120]. By integrating micromachines, researchers can enhance the targeted delivery and activation of these tracers within specific tissues. Click chemistry, known for its high efficiency and biocompatibility, provides an ideal tool for rapidly conjugating radiolabels to targeting molecules or micromachines under mild conditions, ensuring stability and functionality.

**Figure 5:**
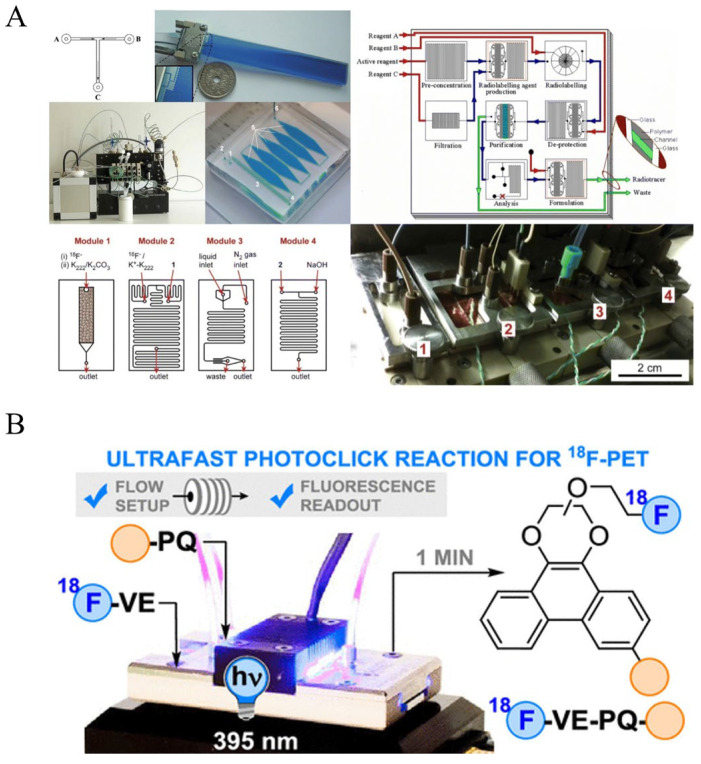
(**A**) Microfluidic platforms for next-generation PET tracer. (**B**) Ultrafast photoclick reaction for selective 18F-Positron Emission Tomography tracer.

In particular the development of microfluidic-based Positron Emission Tomography (PET) [121] tracer synthesis systems has been an active area of research for over a decade [122] (Figure 5A). The unique physicochemical properties of microfluidic systems, particularly their exceptional surface-to-volume ratios enabling rapid heat transfer, have been shown to reduce PET tracer synthesis times by 40–60% while increasing radiochemical yields compared to conventional methods. Furthermore, these platforms demonstrate remarkable reagent economy (using <10% of precursor quantities) and enable automated process optimization. Clinical translation has progressed significantly, with current systems reliably producing doses at therapeutic radioactivity levels while maintaining excellent tracer stability (radiolytic decomposition typically <2% at 4 h post-synthesis).

Click chemistry significantly enhances microfluidic-based PET tracer synthesis by enabling rapid, high-yield radiolabeling under mild conditions through bioorthogonal reactions such as SPAAC and CuAAC, which are perfectly suited for short-lived radioisotopes due to their fast kinetics and exceptional selectivity [123] Ultrafast photoclick reactions enable selective 18F-labeling for PET tracer synthesis in continuous flow systems (Figure 5B), where photochemistry’s spatiotemporal control improves radiochemical yield (>90%) while minimizing side products, critical advantages for producing clinical-grade radiotracers with short-lived isotopes [124].

The combination of click chemistry’s efficiency with microfluidics’ precise control over reaction parameters allows for dramatic reductions in reagent consumption, radioactive waste, and synthesis time while improving the reproducibility and enabling the automated, scalable production of clinical-grade tracers. This synergistic approach overcomes traditional limitations in radiochemistry by facilitating multi-step reactions in compact systems, minimizing purification needs through cleaner reactions, and supporting the development of novel tracers with improved in vivo stability and targeting specificity. The inherent modularity of click reactions further accelerates tracer prototyping and enables flexible integration with diverse radioisotopes and targeting vectors, making microfluidic platforms more versatile for both preclinical research and clinical applications. By leveraging these advantages, click chemistry in microfluidics is transforming PET tracer production into a more efficient, cost-effective, and accessible process while maintaining compliance with stringent regulatory standards for radiopharmaceuticals.

This synergistic combination enables advanced applications such as real-time tracking of drug delivery, precise tumor imaging, and responsive therapeutic activation, paving the way for next-generation diagnostic and treatment technologies in precision medicine.

### 7.3. Biohybrid Micro- and Nanorobot Systems

Nanoparticle-modified microrobots are the latest innovation that combine the accuracy of micromachines with the multipurpose properties of nanoparticles. By integrating nanoparticles into a micro-robot, scientists can significantly improve their functionality and enable this microscopic measurement to perform complex tasks in a controlled and targeted manner. Nanoparticles usually have a variety of uses, including drug loading, imaging, sensing, and advancement through external fields such as magnetic fields or photo stimulation. For example, magnetic nanoparticles can be used to guide microrobots in places in the human body or surrounding environment to achieve precise targeted therapies, such as for cancer treatment or for obtaining strong local drug delivery [125] In addition, nanoparticles with different biological molecules can be functionalized. This allows for biologically specific targeting, such as binding to cancer cell markers. The integration of nanoparticles improves the microrobot’s skills and is very effective in biomedical applications, environmental monitoring, and even materials synthesis. The synergistic effect between microrobots and nanoparticles opens potential opportunities for advances in medical, diagnostic, and minimally invasive personalized operations [126].

The integration of click chemistry with micromachine technology, particularly through the functionalization of nanoparticles, has been explored in several studies, highlighting its potential for precise spatiotemporal control over reaction environments.

Zhang et al. studied the use of biohybrid microrobots in the treatment of targeted anti-cancer (especially lung metastasis) [127]. The research focuses on the development of microrobots that combine living cells, such as bacteria or other microorganisms, with synthetic materials to create hybrid systems that can be accurately and aggressively navigated in the body. These microrobots are designed to carry drug-loaded nanoparticles and are transported specifically to cancer cells in the lungs, significantly improving local treatment for metastatic tumors.

The paper highlights how micromachines, coupled with the use of click chemistry, enable the microrobots to attach drug-loaded nanoparticles at the tumor site with high specificity.

The authors demonstrate that the biohybrid microrobots can actively navigate and respond to environmental stimuli, allowing them to reach lung metastases efficiently. In preclinical studies, these microrobots showed promising results in inhibiting tumor growth, suggesting that they hold great potential for improving the precision of cancer treatments, particularly in the case of metastasis [127].

Qi et al. propose a novel biosensing platform that combines click chemistry with a DNA-based molecular machine to achieve high sensitivity and the absolute quantification of microRNA (miRNA) molecules. In this system, the digital DNA walker (ddWalker) is limited to a single magnetic nanoparticle, which allows for the precise control of its movement and function [128]. The ddWalker works with a number of well-defined click chemistry-driven reactions that can gradually release reporter molecules in response to the presence of target miRNA sequences. This design not only improves the specificity and sensitivity of the assay but also enables the absolute quantification of miRNA molecules, which is crucial for understanding their role in gene regulation and the progression of diseases. In this study, the combination of click chemistry and DNA-based molecular machines represents a significant advancement in the field of molecular diagnostics and provides a powerful tool for the detection and quantification of biomolecules at the single-molecule level.

### 7.4. On-Demand Synthesis and Personalized Medicine

Emerging research in the field of biohybrid microrobots and click chemistry focuses on the integration of the synthesis and biological components to develop systems that enable precise local therapeutic interventions. This innovative approach has the potential to revolutionize personalized medicine by facilitating the on-demand synthesis of customized therapeutic agents directly at the point of care. By combining the versatility of click chemistry with micromachines, these systems can significantly improve the accuracy and efficiency of healthcare and provide individualized patient care at the appropriate time and most urgent locations. In this sense, the study by Qi [128] that presents a DNA-based molecular machine that utilizes click chemistry for precise, on-demand biosensing could be adapted for personalized medical diagnostics.

### 7.5. Self-Learning, Self-Healing, and Self-Evolving Microsystems

The next frontier in microsystems technology lies in the development of intelligent, adaptive systems capable of self-learning, self-healing, and self-evolution [129]. These advancements will enable micromachines and microstructured surfaces to dynamically respond to environmental stimuli, optimize performance in real time, and even repair or reconfigure themselves autonomously. Future microsystems may incorporate machine learning algorithms and neuromorphic computing at the microscale, allowing them to analyze environmental feedback and adjust their behavior [130].

## 8. Conclusions

The integration of micromachines and click chemistry is a rapidly developing field with great potential for chemical synthesis, biomedical applications, and materials science. By overcoming current challenges and the use of new technologies, micromachine-assisted click chemistry could revolutionize multiple industries, from pharmaceuticals to environmental remediation. The continuation of interdisciplinary collaboration will be key to leveraging the full potential of this innovative approach.

## Data Availability

Not applicable.

## Abbreviations

MEMS	Microelectronic mechanical system
CuAAC	Copper-catalyzed azide–alkyne cycloaddition
SPAAC	Strain-promoted azide–alkyne cycloaddition
PDMS	Polydimethylsiloxane
MNRs	Micellar nanoreactors
DTAs	Dendritic amphiphiles
PPFPA	Poly(pentafluorophenyl) acrylate
NIR	Near-infrared radiation
CNC	Carbonaceous nanocalabash
AI	Artificial intelligence
PCL	Polycaprolactone
miRNA	MicroRNA
ddWalker	Digital DNA walker
